# The attitudes and beliefs of general practitioners towards clinical practice guidelines: a qualitative study in Al Ain, United Arab Emirates

**DOI:** 10.1186/s12930-018-0041-2

**Published:** 2018-05-30

**Authors:** Latifa Mohammad Baynouna Al Ketbi, Sana Zein Al Deen

**Affiliations:** Ambulatory Health Care Services, Abu Dhabi Health Services, SEHA, PO Box 81815, Al Ain, United Arab Emirates

**Keywords:** Attitude, Clinical practice guidelines, General practice, Healthcare practitioners, Healthcare systems

## Abstract

**Background:**

The efficacy of implementing practices based on the best evidence is determined by the limitations and preparedness of the structure and processes of the healthcare system as well as healthcare professionals’ (HCP) levels of knowledge and acceptance. Facilitating implementation of such practices also partly depends on HCPs’ attitudes.

**Method:**

We investigate the attitudes and beliefs of four groups of physicians in the United Arab Emirates on clinical practice guidelines (CPGs), with a focus on applying revisions to these CPGs in a different setting than the one in which they were developed, and where no locally developed guidelines exist.

**Results:**

CPGs were the main source of information for revisions. We identified a rising concern in the applicability of the recommendations, which persists due to a lack of locally developed revisions. Other concerns include the pressures of practice management changes and of coping with the rapid development in resources and the growing demand on its use. Some international and government-endorsed CPGs were still accepted as being the best candidates for adoption.

**Conclusions:**

This group welcomes evidence-based practice and is supported by electronic medical records, structured care programmes, and ongoing quality monitoring. Barriers and facilitators of clinical practice guidelines are discussed and thoughts on effective implementation strategies are considered.

**Electronic supplementary material:**

The online version of this article (10.1186/s12930-018-0041-2) contains supplementary material, which is available to authorized users.

## Background

In recent years, using clinical practice guidelines (CPGs) has become a common method of ensuring quality care within healthcare systems. Well-developed guidelines and a commitment of the organization to implement guidelines form a crucial preliminary base to ensure the provision of the best care to consumers. Importantly, the key to success in implementing CPGs is in the hands of the doctors. Their resistance to new interventions is the main obstacle to achieving the intended effectiveness of the interventions [1–4]. Exploring the perspectives of physicians can help guide and support the implementation of CPGs in healthcare systems [5].

In Ambulatory Healthcare Services AHS centres in Abu Dhabi, United Arab Emirates, comprehensive healthcare is offered with heavy emphasis on preventative care. As such, the Department of Health of Abu Dhabi issued preventive care guidelines to facilitate the implementation of numerous national prevention programs such as the Well-Child Program, Cancer Prevention Program, and Cardiovascular Prevention Program [6, 7]. Given the high prevalence of chronic illness and the fact that more than 50% of the ambulatory healthcare encounters were for patients less than 18 years old [8], the other practice improvement guidelines focused on chronic disease and child and maternal health.

To facilitate the adoption of the best practices in healthcare, and to implement these practices, medical services in the Emirate of Abu Dhabi received strong support in the form of technology and medical expertise. In guideline implementation, knowledge is transferred and blended with a healthcare system’s various structures and processes. Particularly, the adaptation and adoption of guidelines are greatly affected by the limitations and availability of certain resources (i.e., technology and medical expertise). Therefore, effectively utilizing advances in the Abu Dhabi healthcare system mandates the exploration of healthcare professionals’ beliefs and concerns about how to implement CPGs, which are regarded as important tools in facilitating the use of best practice.

Because of their value in anticipating reduced variations, improving diagnostic accuracy, reducing costs, reducing harm, and promoting effective treatments in the last two decades, CPGs have become a part of daily practice in all healthcare disciplines and specialties. Nevertheless, for effective CPG implementation to occur, guideline development, and implementation must be rigorous and scientific. Guideline implementation is emerging as a science that requires extensive study to ensure timely and efficient transfer of scientific knowledge and best practices [9].

A study was conducted in Al Ain, United Arab Emirates, that included 817 subjects. It aimed to investigate cardiovascular risk factors [10]. The survey included 817 patients. Physicians participating in the project were asked to treat patients who had significant cardiovascular risks according to the United States’ National Cholesterol Education Program (NCEP) Adult Treatment Panel (ATP) III guidelines [11]. An interesting finding was that although physicians were in the research group and participated in planning and conducting the study, adherence proportion to the guidelines was as low as 45%, with adherence peaking at 70%, across the four participating centres [10]. This study suggests that lack of adherence to evidence-based recommendations is not always due to a lack of knowledge, and it suggests that other barriers need to be identified and addressed. Therefore, we sought to investigate physicians’ use of CPGs, and their attitudes toward CPGs. More specifically, the focus of this action-oriented qualitative research study is to determine the barriers and facilitators of CPG implementation and to determine ways to improve the implementation of CPG recommendations.

## Methods

We employed a qualitative design using six focus groups. Specifically, two groups of family physicians trained in the UAE, western-trained family physicians, and family medicine residents were recruited. These three physician specialty types make up the majority of doctors in the UAE primary healthcare system. Each group comprised four to eight participants, except the western-trained doctors, who were a group of six because very few (25 in total) practice in the city. Furthermore, we selected these three populations to achieve some degree of representation of the actual population of practicing doctors and to obtain results reflecting a variety of experiences and perspectives. The focus group approach was used to facilitate generation of opinions and ideas through participants’ interactions and reflections.

The study was approved by Al Ain Human Research ethics committee.

### Participant and focus group procedures

The authors are from the AHS academic affairs department who oversees continuous professional development and practice improvement and the authors interact with Health care centres for education and quality improvement projects. As such, we recruited physicians who their centres believed would be vocal about their experience with CPGs. Furthermore, we recruited physicians who we believed were active participants in their professional development. They were invited from several AHS centres from within Al Ain city. All participants had to meet the inclusion criteria of being a practicing family physician or general practitioner of Ambulatory Health Care Services of the UAE College of Medicine, with at least 2 years of experience in their role.

The 25 respondents were mostly female (16 females and 9 males). Of these participants, eight were family physicians, eight were board-eligible family medicine residents, and nine were general practitioners who had been in practice for more than 15 years. All members of the resident group were female. The other groups accurately represented the population of practicing physicians in the AHS (see Table 1).

**Table 1:** Characteristics of participants

Gender	
Male	9
Female	16
Age	
< 30	7
30–40	12
> 40	6
Clinical qualifications	
Board certified	10
Non-board certified	8
Under residency training	7
Type of practices	
General practice	8
General practice and faculty in residency program (post-graduate)	4
General practice and faculty in College of medicine (under-graduate)	6
General practice and under-training in residency program	7
Years in practice	
< 5	7
5–10	8
10–15	4
> 15	6

Selected participants were invited to the focus groups, which were conducted at the Ambulatory Health Services (AHS) Academic Affairs building. The focus groups lasted from 90 to 120 min. All focus groups were audiotaped and transcribed verbatim by research assistants, who was also a nurse, immediately following the meeting. Data collection proceeded until saturation was reached (Additional file 1).

The first author (LMBK), who holds an advanced degree, conducted all of the focus groups. This author led the focus group and used a guide to run the focus groups. Evidence-based medicine and barriers to the implementation of the key recommendations of two CPGs were discussed. More specifically, the following topics were explored: using CPGs, trust in CPGs and evidence-based medicine, how the guidelines influenced the professionals or clinical practice, what factors facilitated implementation, and barriers to using CPGs. Four CPG recommendation talking points were offered as examples to elicit participants’ opinions and attitudes towards CPG use. Focus group questions targeted the depth of participants’ perceptions and experience. To induce a greater depth of information from subsequent focus groups, questions were redirected based on the information that emerged after each focus group, and this information was used to update the guide.

The data were analysed using grounded theory analysis [12], which focuses on deriving conceptual categories from studying and critically reviewing all collected data. All transcribed lines were read and coded, and then they were organized and grouped into categories based on concepts. Using supporting quotes from the transcripts, themes were then developed from these categories. Both manifest and latent content analyses were performed. In the manifest content analysis, the written words directly expressed in the extracted text were used. In the latent content analysis, the aim was to find the underlying meaning in the text [13].

## Results

A summary of the overarching themes is presented in Table 2. We present the details and select quotes from the focus groups in the following passages to help shed light on this important topic.

**Table 2:** Attitude towards clinical practice guidelines

Theme	Statements
Positive attitude towards guidelines	
Provide evidence-based recommendation	‘Most known guidelines contain summary of all studies and analyses; so I do not have to go through information in parts’ ‘Because it is supported by evidence from many trials and medications’. ‘Recommendations are based on trials that prove its effectiveness; this is more beneficial than the non-trial ones. It is a logical approach’
Cost-effective	‘It’s cost-effective because it is the best care given’
Save time	‘I think we need less time if we know the investigations to be done. It will not take time’ ‘Save time, more comfortable, more convenient. If the physician is aware of the guidelines, it will not take time’
Standardize care	‘It is to standardize the language we speak and health requirements. Like any other business, it is measurable’ ‘More suited to patient’ ‘Trackable care’ ‘Measurable care’
Negative attitude towards guidelines	
Changing evidence	‘The CPG will be behind new studies by six months to 1 year; so we can’t think that it represents the latest evidence’
Contradicting recommendations	‘There are some differences from American associations and others. Some say that HBA1c is a diagnostic test; others say it is a follow-up test’
Lack of ability of the doctors to read EBM	‘You cannot be sure unless you learn how to access the paper and decide whether it is weak or strong. At the same time, there should be guidance from the organizing body on how to work around gaps; there should be some reference for people to go to. As an academic, this what I say but as a physician it is not practical; even the ones who know how to analyse an article, do they actually do it? I don’t think so’
Not applicable to each individual patient	‘Individualized treatment. Guidelines don’t fit each individual’ ‘We can take the basic things and the rest can be tailored for each patient. Not every patient has the same case and same treatment’
Multiple sources	‘Which guideline should you follow? Take this one or that? The British, American, or European’
Transferability of guidelines to local setting	‘All adapted’ ‘Because we don’t have another option’ ‘We think it is true for particular circumstances, for that culture’

### Attitude towards EBM and CPGs

Participants referred to CPGs in their daily work, and expressed an intention to practice evidence-based medicine (EBM). Table 2 provides a description of how EBM recommendations are valued by the participants. Reasons for the favourable opinions included the fact that the sources of evidence were clinical trials, and that using EBM reduced costs and improved efficiency, particularly in terms of time. Providers also tend to use CPGs because they allow for measurable outcomes and tracking of progress over time.

Although many opinions of CPGs were favourable, some participants expressed a negative attitude toward CPGs, citing conflicting recommendations in different guidelines, the presence of an unwieldy number of guidelines, and changing evidence. This group of individuals mentioned taking caution when implementing CPGs, and they also noted the importance of tailoring treatments to the individual. Finally, those in opposition to CPGs stated that they were concerned that none of the guidelines were developed locally, and this led to concerns about the validity across cultures.

### Adapting CPGs

One of the family physicians in our study expressed concerns regarding adapted CPGs, CPG developed in other country and modified for their new setting, (see Table 3), calling it a “risk” since it was developed for other setting. However, other participants found that using CPGs from multiple sources offered an expanded knowledge base.

**Table 3:** Opinions about the sources of CPGs and the use of locally adapted ones

Theme	Quotes from participants
Sources of CPG used	‘Most famous, trustable, acceptable by the community or you as a reader’ ‘Mostly updated’ ‘Applicable to patient’ ‘Should be from recognized body; not from just anywhere’ ‘No drug company involvement’ ‘Be government-funded’ ‘Should answer queries’ ‘Origin of guideline’ ‘Supported by organization’ ‘It depends on how the guidelines present the information’
Different culture and patients’ population	‘I will take the guidelines because it is updated but in my opinion, patients differ here from the UK and USA’ ‘Adapted guidelines are trustworthy and I will not hesitate to choose [them]’ ‘We think it is true for particular circumstances, culture, and politics. We have to modify and produce our own practice [guidelines] and we have to conduct research’ ‘We are using it because we don’t have another option’ ‘It is successful [but] we cannot copy and paste all the time. We need information from our community and the problems we are facing’ ‘You can take what you need, and you can be selective according to the community and patients’ beliefs’
The ability to be selective and use the best knowledge from different CPGs	‘[You can] combine more than one guideline to find all information needed’ ‘The volume of information is more in the original [guidelines]; local guidelines include only the useful information and applicable ones’ ‘It is easier, as the American Diabetic Association contains all the details and as a family physician I don’t need all that information; it is useful to know but it is too detailed’
Being endorsed by the institution	‘Our guidelines adopt the most recent guidelines’ ‘Adapted guidelines have the power of authority of the local organization’
Perceived risk	‘Risk, there should be standards or rule to follow any miss- phrasing can lead to wrong information’, ‘Should be ethical and mention the source’, “Not biased to any area, experience or need, we should mention all drugs and institution should follow recommendation”, “Self breast exam is harm but it is still in the national program and I am not following”, “We have to raise it up, they have something in their mind”, “We don’t know who is putting it, the things that supposed to be removed should be referred by special person whom we don’t know”, “We don’t know the methodology, partially we are not relying on the organization guidelines and in other parts where we are sure they are true we are relying on them”
Guideline representation	‘Customize the international guidelines to become national guidelines’ ‘Easier’, ‘Shorter’, ‘Relevant parts only’, ‘Simple’, ‘Easy language’, ‘Practical effective parts’

There was a consensus among participants that adaptation of CPGs should be performed by the institution or a government organization.

### Sources of CPG

The CPGs referenced by participants were all international CPGs, or they had been adapted from international CPGs. Examples that were provided to participants for review were the Scottish Intercollegiate Guidelines Network (SIGN) asthma guidelines [14], the National Institute for Health and Care Excellence [15] diabetes mellitus guideline, and the Institute for Clinical System Improvement [16]. All of the guidelines were accessed through the local institution’s e-library or they were disseminated by the Health Authority Abu Dhabi. The CPGs were mainly communicated through Continuous Medical Education (CME) workshops and email.

### CPGs’ barriers and facilitators

To assess attitudes towards implementation of CPGs, participants were given CPG recommendations and then they were asked about their agreement with each, as well as their intentions for implementation. Tables 4 and 5 provide detailed information about attitudes, barriers, and intentions with CPGs.

**Table 4:** Barriers identified for known recommendations

	Condition-related	Patient preferences	Medication or prescribing related	Test or test ordering related	Lack of continuity of care	Doctors knowledge or experience	Insurance related	Lack of structured care	CPG recommendation	Doctors’ perceived feasibility	Time factors
OGTT as screening test for pregnancy		•		•	•					•	
Prescribing lipid lowering agents		•	•		•	•	•	•	•		•
Action plan for asthma		•			•	•		•		•	•
Osteoporosis screening	•					•		•		•	•
Self breast exam						•			•		
Nephropathy screening in diabetes				•	•	•			•		
Aspirin use in diabetes			•			•			•

**Table 5:** Barriers and facilitators of CPG use

Themes	Quotes on perceived effective implementation strategies
Barriers	
Insurance coverage of services	‘Insurance does not cover the drug’
Competition of private sector	‘Continuity of care, the private clinics does not have guidelines’
Patient-related	‘Patients’ acceptance’ ‘They don’t like to break their fast on Ramadan days’ ‘The taste of the oral solution’ ‘A lot reject the test’/‘They vomit’ ‘1 in 4 will accept’
Doctor-related	‘Patients are not coming’ ‘Asthma action plan is devised by the chest physician’ ‘It (asthma action plan) will take time from doctors’ ‘Doctors believe and practice’ ‘Doctors are interested; we are checking the KPI and commenting on how to improve the practice’
Communication between hospitals and AHS	‘It is followed in the hospital’
Lack of structured care for some conditions (e.g., asthma and osteoporosis) compared to widely implemented structured care for diabetes and hypertension in the AHS	‘You have to choose the ones who are interested. You should not choose all. Doctors who don’t care shouldn’t be in the institution’ ‘Most have their spirometer but some clinics don’t’ ‘Accessing the whole organization and not individuals’ ‘It differs if you have a chronic disease care clinic. Doctors will be under pressure by other patients and will not give good care, and some doctors don’t have a sense of responsibility’ ‘There are no guidelines for osteoporosis’ ‘No, it is not like diabetes mellitus (DM); there are no guidelines and no special clinics’ ‘We are not following our target patients (osteoporosis patients)’ ‘It is a mistake of the institutions to not recommended screening for adults’ ‘Having well women clinics is better than having GP clinics’
Condition-related	‘There is a higher prevalence of DM, complications, and diagnosis’, ‘easier to diagnose DM’, ‘all age groups have DM’
Facilitators	
Accessibility of knowledge in the office	‘Makes things easier; so, if you have any questions you have the answer easily’ ‘It reduces the anxiety of feeling alone, especially during out-of-hours clinics’ ‘Calculators are available in computers and programmes’
Quality monitoring	‘Auditing’ ‘Institutional KPI’ ‘Patient satisfaction KPI’ ‘Guidelines improve their KPI; it should support the KPI or targets’, ‘They are seeking the KPI level four times per year’ ‘Other types of auditing, which we don’t know about in hospitals, like how our care affects admissions, complicated patients, and compliance’ ‘Yes, now they are trying their best to better achieve the KPI’ ‘To reach the KPI and help patients’
Endorsement from the institution	‘They formulated guidelines but didn’t work to improve implementation of guidelines…it is individual work’ ‘If the guidelines are available in the institution, it is the responsibility of all to follow it because we all care for the same patients and we should speak the same language with the patient’ ‘About breast cancer screening; it is a national programme. They didn’t give the option to do it or not. So, we are applying it and until they change it I have to follow it as it is supported by the organization’ ‘We cannot follow the institution always; this depends on the situation because if what is recommended by the institution is wrong we might miss-practice and put the patient at risk’ ‘As long as the guidelines are issued by the organization it is more likely to be followed and more likely that they have something in their mind; we are not aware of all statistics they have. They have all statistics and information, and as long as it is not harmful we follow them’ ‘The HAAD and SEHA are looking for quality now’
Electronic medical records	‘It is difficult with paper medical records and needs staff’ ‘Introduction to m-pages (health maintenance reminder page) is one way of helping people to follow the guidelines’ ‘If it used, it is effective’, ‘guidelines link to medical records’
Structured care	‘It differs if you have a Chronic Diseases Clinic from if you don’t, and doctor will be pushed by other patients and will not provide good care. Some doctors don’t have a sense of responsibility’ ‘If not, Chronic Diseases Clinic performance will be the same? I don’t think [so] at all’ ‘If I was a GP and a chronic disease patient visited me, I will not be able to attend to him well, because many more patients will be waiting outside’

Table 4 details the perceived barriers to implementing CPGs for well known accepted care recommendations reported by family medicine practitioners. The cited barriers were related to the patients’ condition, patient preferences, medication or test characteristics, practice settings, physician knowledge, payment systems, related recommendations, feasibility or physician-perceived feasibility, and time factors. All of these barriers must be considered in the planning implementation steps. The perceived barriers may vary based on the demands of the clinical situation. For example, the barriers in guideline recommendation may be very low when a provider is ordered a mammogram to screen for breast cancer versus when the provider is managing a complex patient with dyslipidaemia.

In addition to barriers to CPG implementation. Participants also identified a number of important facilitating factors. First, participants noted that electronic medical records (EMRs), and having easy access to computers within offices, help with facilitating CPG implementation. Overall, while participants noted some barriers to using EMRs, including perceived burden due to documentation requirements, they felt that this practice would facilitate CPG implementation. Second, organizational endorsement and quality monitoring were also noted as strong facilitators of implementing the CPGs. Third, structured care programmes, in particular those led by a central committee who supervise and conduct ongoing training of teamlets (a tightly knit group with one clinician and one or two assisting professionals working together closely) in all AHS centers, are perceived as being effective facilitators.

## Discussion

### Attitude towards CPGs

All participants in the present study had existing knowledge of CPGs, and they considered CPGs fundamental for their practice. Participants’ concerns about CPGs were similar to those reported in previous studies, and include conflicting recommendations from different guidelines, changing evidence, and the lack of generalizability of most recommendations. There were also concerns about the need to individualize implementation. In contrast with the views of the participants in the present study, Carlsen et al. reported that the changes in recommendations and disagreement between experts are mainly viewed as positives because of changing knowledge and different interpretation and implementation prospective [17, 18].

Although participants in our study viewed CPGs as being fundamental for practice, participants did report a number of barriers. This is consistent with previous research which found that among Belgian social insurance physicians, knowledge of EBM and CPGs was rather poor, and perceived barriers for applying evidence to practice were mainly time and lack of EBM skills [19]. Taken together, this information suggests that adopting and implementing CPGs involves multiple variables, and physicians who are supposed to implement these guidelines may have variability in their training which further affects their ability to implement and evaluate CPGs.

### Adapting CPGs

Participants generated a number of factors which they perceived as limiting implementation of CPGs. A strongly stressed and unique concern (to the point where it was considered a risk) was the notion that practitioners were implementing CPGs designed based on external research done in different, possibly incompatible, populations. Endorsement or adaptation of the CPGs by a governmental body was effective in reassuring providers that it was acceptable to use these recommendations. Some participants commented that there is freedom in implementation of guidelines; however, other have argued that combining guidelines, or following them in a piecemeal manner can result in confusion or deficient implementation. As such, adapting CPGs remains a challenging task, especially for organizations in countries with limited research data and scarce locally-developed CPGs. Consequently, this requires practitioners to make careful decisions either to use caution when implementing and adapting CPGs across cultures.

The Institute of Medicine defines CPGs as “statements that include recommendations intended to optimize patient care that are informed by a systematic review of evidence and an assessment of the benefits and harms of alternative care options” [20]. When guidelines are revised and adapted in order to fit different cultures, it is important for adaptation to be conducted by individuals with credentials and experience similar to the developers of the guidelines. Undoubtedly, cross-cultural research area has started to attract greater interest [21].

### Common facilitators and barriers

The other barriers to implementation we found share some similarities with those identified in a review of barriers to guideline implementation by general practitioners (GPs). Six categories of barriers were identified: the content of the guidelines, the format of the guidelines, GPs individual experiences, preserving the doctor–patient relationship, professional responsibility, and practical issues [17]. Similar barriers were found by other researchers as well [18, 22, 23].

The fact that disease-specific facilitators and barriers in CPG implementation exist suggests that physicians implementing CPGs should be mindful of the disease to be managed as well as the clinical setting. Unfortunately, there is no single solution for all to be successful in implementing and adhering to best practices. When suggesting example recommendations and challenging participants with different barriers and facilitators for each recommendation, we noticed that not all recommendations followed a similar path for implementation. A unique implementation plan should be tailored with frequent review of all possible barriers and facilitators as a means of reaching the optimum outcome, especially when implementing these guidelines with diverse populations. Barriers identified by our groups highlight the challenge in reconciling findings from well-controlled studies with realistic clinical environments. The complexity of patients and their beliefs, economic burden, medication efficiency, and side effects contribute to this challenge. For example, recommending ordering diagnostic test as mammogram or prescription of Aspirin have far less time and cost implications than on working on asthma action plan with chronic asthmatic patient. Probably the later needing more time, counselling skills and knowledge but more importantly as highlighted by the group needing supportive health care system design through continuity and structured care.

This calls for changes in implementing the best evidence. Indeed, developing and disseminating guidelines is only part of the process of ensuring that these CPGs are implemented appropriately. Careful implementation, with subsequent quality checks are needed. Those who choose to implement the guidelines also need to be particularly aware of the cultural environment in which they practice. In particular, ongoing monitoring using performance indicators that include patient satisfaction and outcomes are encouraged.

Dissemination methods described by the participants in the present study are similar to those in other studies. In particular, we found that institutions seem to be essential in disseminating evidence. Institutions can be seen as powerful agents to improve care. Participants reported that they would prefer their institution be the source of the CPG dissemination. Furthermore, all participants were committed to using any available tools to help improve their outcomes.

Our results supported a need and desire for a multifaceted approach to implementation of CPGs. In particular, participants noted the importance of patient education and empowerment, healthcare professional education, practice change, and resource provision. All of these were highlighted as perceived challenges of this group of participants.

The attitude towards EMRs was positive. EMRs were perceived as a strength and opportunity to facilitate EBM. A growing number of studies have reported the role of EMR in facilitating evidence-based practice [24, 25].

The structured care programme for chronic diseases, which was used in all participants’ workplaces, was highly valued by participants as a means of helping them to employ best practices. This is not a surprise, as the components of the Ambulatory Healthcare Services (AHS) chronic disease programme are based on the chronic disease model, and interventions and tools used in the model are seen by the group as facilitators. Facilitators included daily structured clinics, reminders, outcomes, self-management programmes, educational activities for the Health Care Professionals (HCP), facilitated team communication, and continuous dissemination of new updates in email communications. Others included meeting with chronic disease champions and coordinators and allowing for feedback from providers [26].

The present study is not without limitations. The participants did not indicate the means by which they learned of CPGs. In future research, participants’ sources of knowledge need to be explored in greater depth. One limitation could be participants’ tendency to agree with the group norms. Although we took multiple steps to facilitate expression of different opinions by asking questions in different formats, and by using props to ensure understanding and depth, it is possible that group normative pressures interfered with the ability to fully express opinions.

Another limitation of this study is the fact that we used self-report measurements of CPG use and knowledge, which are best assessed using other methods, including knowledge assessment and practice measurements.

## Conclusions

The insight of the groups on effective strategies for implementing best evidence through CPGs reflects the strong institutional environment provided by an implanted EMR structured care programme and ongoing quality monitoring. Our results highlight areas of importance in delivering the best evidence in this setting through greater structure and governance of adapting CPGs. In addition, the results showed that participants valued the encouragement of local clinical research, which can improve these processes, as well as health service research, which can help them utilize the resource-welcoming environment of evidence-based medicine.

## Additional file

**Additional file 1.** Focus group discussion moderator’s guide including questions and probing statements used.

## Abbreviations

EMR: electronic medical records
CPGs: clinical practice guidelines
NCEP: National Cholesterol Education Program
ATP: Adult Treatment Panel
UAE: United Arab Emirates
AHS: Ambulatory Healthcare Services
EBM: evidence-based medicine
SIGN: Scottish Intercollegiate Guidelines Network
CME: Continuous Medical Education
