# Supplementary material for: The attitudes and beliefs of general practitioners towards clinical practice guidelines: a qualitative study in Al Ain, United Arab Emirates
Source: Asia Pac Fam Med. 2018 May 30;17:5. doi: 10.1186/s12930-018-0041-2 (PMC5975561; doi:10.1186/s12930-018-0041-2)
Supplement: Supplementary file 1 — Additional file 1. Focus group discussion moderator’s guide including questions and probing statements used. [file 12930_2018_41_MOESM1_ESM.docx]

Focus Group Discussion

Moderator’s Guide

INTRODUCTION**: Hello. My name is ___________ and I’m the moderator for today’s 60 minute group discussion/interview. ____________ will be taking notes. Our purpose today is to:**

1. **Firstly, get your insights and understanding about the Clinical Practice Guidelines and Evidence Based Medicine?**
2. **And learn what are the best tools to practice evidence based medicine and to answer questions you encounter in your practice?**
3. **Secondly, get your insight and understanding about Clinical Practice Guidelines and your opinion about its use in your practice and if you think it is a helpful or not and if helpful barriers and facilitators of its use?**
4. **And learn about your views on the use of specific Clinical Practice Guidelines?**
5. **Thirdly, get your views and suggestions to implement best practice.**

### SELF-INTROS:

**Please introduce yourself and tell us:**

- **Your name, year in practice and qualifications.**
- **Your name, responsibility, clinic/department.**

This discussion is one of a series being held as part of a research approved by the Al Ain Human Ethics Committee (32/11) aiming to improve the use of clinical practice guidelines.

Feel free to make any negative or positive comments about any of the things we will be discussing today. This is a free flowing discussion and there are no right or wrong answers. Before we get started, here are some ground rules and points of information:

### DISCLOSURES:

- CONFIDENTIALITY. Everything that you say here will be kept strictly confidential. Nothing said in this group/interview will ever be associated with any individual by name. We would also ask that you similarly maintain the confidentiality of what is said in the group/interview.
- VOLUNTARY PARTICIPATION. Your participation in this group/interview is entirely voluntary. You may stop participating at any time. You do not have to respond to any questions that you do not wish to answer. You may withdraw from the group/interview at anytime with no consequences. The consent forms provide more detailed information regarding confidentiality and the voluntary nature of participation. If you haven’t already done so, please sign the consent form and pass it to __________.
- AUDIO-TAPING. This session is being taped so that we can write an accurate report-not of who said what. If there are any objections we will not tape the session.
- THANKS. Thank you for arranging your schedule today to be here for this session. We really appreciate you giving us your time, opinions and courage.

### GROUND RULES:

### For Focus group:

1. Please talk one at a time in a voice as loud as mine.
2. Avoid side conversations with your neighbors.
3. We need to hear from everyone in the course of the discussion, but you don’t have to answer every question.
4. Feel free to respond directly to someone who has made a point.
5. You don’t have to address your comments to me to get them on the table.
6. Say what is true for you and your situation and have the courage of your conviction. Don’t let the group sway you and don’t “sell out” to group opinion.

**For individual interview:**

1. Please talk loud and clearly.
2. Be as explicit and honest as possible in your views. Say what is true for you and your situation and have the courage of your conviction.

# Questions and probing statements:

I would like to start with asking:

1. Do you think practicing evidence based medicine is the right choice and why?
2. What are the best tools to practice evidence based medicine?
3. How to answer questions you encounter in your practice?
4. Why Clinical Practice Guidelines CPG?
5. Is clinical practice CPG in your opinion represents the “scientific evidence”?
6. From where you get the guidelines? SOURE
7. How you decide to use a guideline? WHICH to USE?
8. What are the important attributes of clinical practice guidelines?
9. Should CPG be followed always? When you think about applying the CPG what are the important barriers and facilitators?
10. Can CPG developed elsewhere be implemented in Al Ain? Is there extra dimension here?
11. What we mean by adapting the guidelines? Is it good or can it be bad? Should we have standards to who and how to adapt guidelines?
12. Your experience with CPG adapted locally? Compare 2 i.e. ADA and local DM , Asthma four CPG?
13. Who should advice or adapt the CPG?
14. Is the organization you are working at important for the CPG choice and adaptation and implementation?
15. What you think about the following recommendations and its experience in being implemented in Al Ain? Do you agree with it? Is it feasible? Applicable? Barriers? Facilitators?
16. OGTT use for the screening of Gestational Diabetes
17. Diabetes Mellitus management
18. Aspirin for Primary prevention
19. Asthma action plan for asthma self management
20. Screening and Prescribing lipid lowering in Dyslipidemia
21. Osteoporosis screening
22. Breast cancer screening
23. What you think about CPG influence on cost, patient care, and service?
24. How to improve knowledge about CPG and knowledge about its content?
25. Are CPG important for your learning?
26. How is important in your learning? The last updated information you knew it from where?
27. From where comes most of your learning? As individual , at home or as a group or at office?
28. How to improve use and implementation of guidelines? Did moving electronically improved knowledge and use?
29. How to ensure CPG recommendations are implemented? What strategies you suggest?
30. If we have almost the best resources in AHS as you mentioned, why KPI results are not the best?
31. If you think CPG needs to be adapted content wise on what bases? How to decide to change content?
32. On what bases we must adapt the CPG? How to build local data?
